# Hybrid Finite Element–Smoothed Particle Hydrodynamics Modelling for Optimizing Cutting Parameters in CFRP Composites

**DOI:** 10.3390/polym15132789

**Published:** 2023-06-23

**Authors:** Alessandro Abena, Sabbah Ataya, Hany Hassanin, Mahmoud Ahmed El-Sayed, Mahmoud Ahmadein, Naser A. Alsaleh, Mohamed M. Z. Ahmed, Khamis Essa

**Affiliations:** 1 School of Engineering, University of Birmingham, Birmingham B152TT, UKk.e.a.essa@bham.ac.uk (K.E.); 2 Department of Mechanical Engineering, Imam Mohammad Ibn Saud Islamic University (IMSIU), Riyadh 11432, Saudi Arabia; smataya@imamu.edu.sa (S.A.); naalsaleh@imamu.edu.sa (N.A.A.); 3 School of Engineering, Technology, and Design, Canterbury Christ Church University, Canterbury CT11QU, UK; 4 Department of Industrial and Management Engineering, Arab Academy for Science, Technology and Maritime Transport, Alexandria 21599, Egypt; dr.mahmoudelsayed12@gmail.com; 5 Department of Production Engineering and Mechanical Design, Tanta University, Tanta 31512, Egypt; m.ahmadein@f-eng.tanta.edu.eg; 6 Mechanical Engineering Department, College of Engineering at Al Kharj, Prince Sattam Bin Abdulaziz University, Al Kharj 16273, Saudi Arabia; moh.ahmed@psau.edu.sa

**Keywords:** finite element modelling, smoothed particle hydrodynamics, orthogonal cutting, chip formation

## Abstract

Carbon-fibre-reinforced plastic (CFRP) is increasingly being used in various applications including aerospace, automotive, wind energy, sports, and robotics, which makes the precision modelling of its machining operations a critical research area. However, the classic finite element modelling (FEM) approach has limitations in capturing the complexity of machining, particularly with regard to the interaction between the fibre–matrix interface and the cutting edge. To overcome this limitation, a hybrid approach that integrates smoothed particle hydrodynamics (SPHs) with FEM was developed and tested in this study. The hybrid FEM-SPH approach was compared with the classic FEM approach and validated with experimental measurements that took into account the cutting tool’s round edge. The results showed that the hybrid FEM-SPH approach outperformed the classic FEM approach in predicting the thrust force and bounce back of CFRP machining due to the integrated cohesive model and the element conversion after failure in the developed approach. The accurate representation of the fibre–matrix interface in the FEM-SPH approach resulted in predicting precise chip formation in terms of direction and morphology. Nonetheless, the computing time of the FEM-SPH approach is higher than the classic FEM. The developed hybrid FEM-SPH model is promising for improving the accuracy of simulation in machining processes, combining the benefits of both techniques.

## 1. Introduction

Fibre-reinforced polymers (FRP) are currently experiencing a growing demand across various industries, including automotive, aerospace, construction, and marine. The reason for this is the remarkable characteristics of these materials, which include particular strength and stiffness, damage resistance, resistance to fatigue, corrosion, and acoustic and thermal insulation properties, all of which surpass those of metals and ceramics [1]. Since the 1980s, composites have been widely used in several applications and currently constitute about 50% of the entire structure of modern aircrafts [2,3]. Due to this demand, aerospace manufacturers invested in developing FRP materials and the complemented machining process [4]. Machining procedures such as turning, milling, drilling, and deburring are essential in CFRP production to achieve specific geometric tolerances and create holes for fastening during the assembly process [5]. Although the machining of traditional materials is well established, the machining of composites such as CFRP is still in its early stages, and the interaction between tool and workpiece requires further investigation. Machining CFRP poses several challenges, such as the high wear rate of cutting tools and fibre delamination during cutting [6].

Understanding chip formation, tool–workpiece interaction, and material behaviour in composite machining requires extensive and costly experimental work. Advanced equipment is needed to analyse the process at a microscale level, which increases expenses. Alternative methods include empirical, analytical, and numerical techniques. However, the empirical method relies on limited experimental measurements, providing limited information without insights into material failure and chip formation. Analytical techniques also fall short of accurately representing process intricacies [7]. The FEM is commonly used for machining analysis, with ongoing efforts to improve its accuracy for simulating CFRP machining. A micro-mechanical approach can be employed to simulate each composite phase individually, allowing for a detailed assessment of material removal and damage. However, the accuracy of this technique depends on assumptions and material data availability [8].

Limited research exists on analytical studies of FRP orthogonal cutting. Researchers have adapted Merchant’s shear plane theory for FRP machining, but it has limitations. Takeyama and Iijima [9] developed a 2D FRP machining model based on this theory, but it disregards temperature effects and is limited to FRPs with fibre orientations below 90 degrees. Zhang et al. [10] used a 2D FEM model to analyse cutting tool geometry effects, identifying chipping, pressing, and bouncing regions. FEM simulation of composite machining is categorized into micro-mechanical, macro-mechanical, and micro–macro-mechanical approaches [4,10]. The macro-mechanical approach assumes FRPs as homogeneous materials and calculates their properties using the rule of mixtures [11,12,13]. In contrast, the micro-mechanical approach uses finite element method (FEM) models to work on each phase of the FRPs separately, providing a better understanding of material failure and chip formation during machining and enabling more detailed simulations [9,14]. However, this approach requires high computational resources and is not suitable for complex machining models [15,16,17,18]. To address this issue, researchers have developed the meso-scale approach, which employs micro-modelling near the cutting tool while using the homogenized material approach for the rest of the material, resulting in reduced computational costs and providing insights into machining FRPs [19,20].

To achieve accuracy to simulate failure and removal of materials during machining, material models must incorporate specific stiffness degradation and failure modes [21,22,23,24]. Many studies have focused on composites with carbon or glass fibres (GF) with a polymer matrix. The mechanical properties of the polymer matrix are influenced by temperature, rate of strain, and loading, and are typically presented as an elasto-plastic curve using isotropic hardening and Von Mises stresses. The degradation in material stiffness is incorporated until material failure occurs [25,26]. On the other hand, carbon fibres exhibit isotropic properties that are typically independent of the rate of strain [27,28], while GFs that depend on strain rate are isotropic [22]. Researchers have conducted limited experimental studies to obtain the necessary fibre mechanical properties for FEM analysis [29,30,31]. Dandekar and Shin [32] used the Marigo concept to express the brittleness failure of CFRP. On the other hand, Rao et al. [21] applied transversely isotropic properties and a maximum principal stress failure criterion, which resulted in significant discrepancies between FEM and actual measurements of the thrust and cutting forces [23].

To develop a model that describes the bonding mechanism between fibres and the matrix in composite materials, researchers commonly use a cohesive zone model. For instance, Santiuste et al. [12] utilized this model in a macro-mechanical approach to analyse failure due to out-of-plane deformation of long fibre-reinforced polymer (LFRP) machining [33,34,35]. In a separate study, Camanho and Davila proposed a mixed-mode de-cohesion element to simulate the interface between elements and to model the initiation and growth of delamination [36]. Furthermore, May et al. [37] developed a strain-rate-dependent model, while Salih et al. [38] incorporated an elasto-plastic model into the constitutive equations.

Smoothed particle hydrodynamics (SPHs) is a meshless numerical approach that has gained popularity in recent years due to its ability to overcome the limitations of discretization in FEM in applications such as fluid mechanics, solid mechanics, and multi-physics problems [38,39,40,41,42]. Several studies have employed SPHs to simulate the machining of metals. Limido et al. [43] developed a 2D model for orthogonal cutting, realizing that the SPH method is more capable of representing the actual separation between the chip and the workpiece. Villumsen and Fauerholdt [44] studied the effects of particle resolution, mass scaling, and time scaling on the cutting force, while Avachat and Cherukuri [45] conducted a parametric study on the influence of machining process parameters on workpiece quality. Xi et al. [46] utilized a hybrid FEM-SPH approach to investigate thermally assisted machining of Ti6Al4V, revealing the development of chip formation and cyclic cutting forces during machining. However, current studies on machining typically use 2D plane strain orthogonal machining models, which accurately predict cutting forces but inaccurately estimate the thrust forces. Although machining processes are typically three-dimensional, few studies have incorporated 3D machining models [47]. These studies’ limitations are that they have only modelled a portion of the workpiece, resulting in an inability to predict critical physical parameters such as stresses, strains, and temperatures accurately.

Conventional approaches to simulating orthogonal cutting of CFRP have limitations. Empirical methods require experimental calibration and provide limited predictions. Two-dimensional FEM models focus on chip formation, while local information requires detailed material data. Micro-mechanical approaches using zero-thickness cohesive elements can be distorted and lead to poor thrust force prediction. To overcome these challenges, this paper introduces a novel FEM-SPH hybrid algorithm. It combines the strengths of both methods by addressing material loss with SPHs and implementing a cohesive interface model for the matrix–fibre connection. This allows for information collection at the interface, which was previously not possible. The proposed method is compared with a 3D FEM simulation and validated through experiments to assess chip formation, thrust, and cutting forces.

## 2. Modelling and Experimental

### 2.1. Smoothed Particle Hydrodynamic Model

In this approach, the workpiece being machined is comprised of spherical particles. The estimation of the summation form of a property for a given particle subject to a non-zero kernel function, W, was determined as follows:

(1) $$f\left(x\right)≅\underset{j}{\sum }\frac{m_{j}}{\rho_{j}}f_{j}W\left(\left|x-x_{j}\right|,h\right)$$

**Figure 1:**
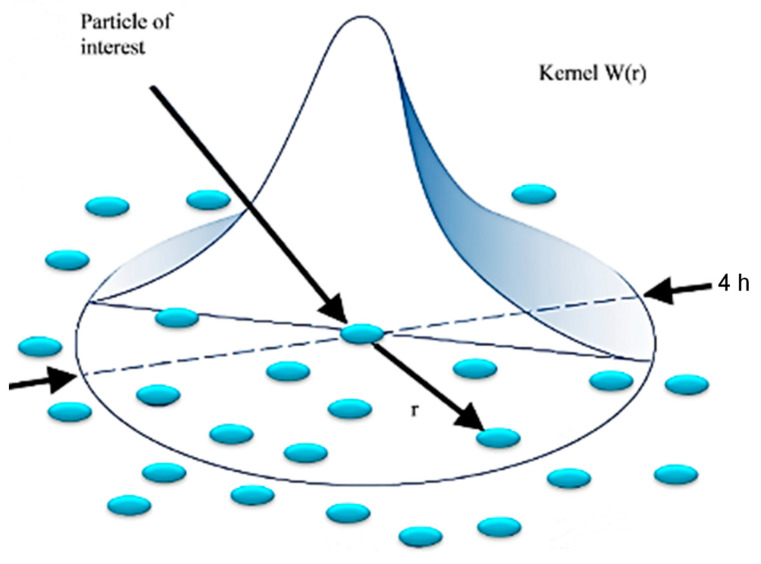
A representation of kernel (W) function in the smoothed particle hydrodynamic model.

The variables in the SPH method consist of mass (*mj*), density (*ρj*), location (*xj*), and the neighbouring particle *j* variable (*fj*). The smoothing length coefficient (*h*) determines the sphere of influence of the kernel function, *W*, which is responsible for computing the variable of interest based on neighbouring particles. This is demonstrated in Figure 1. Similar to the FEM, the implementation of a constitutive model is required to govern the interaction between particles.

The smoothed particle hydrodynamic (SPH) method is effective for simulating material openings caused by tool action. Unlike FEM, SPHs does not require the removal of failed particles but accommodates material property degradation. However, the lack of a cohesive model in SPHs limits the collection of information at the fibre–matrix interface.

### 2.2. Hybrid Model Development

**Figure 2:**
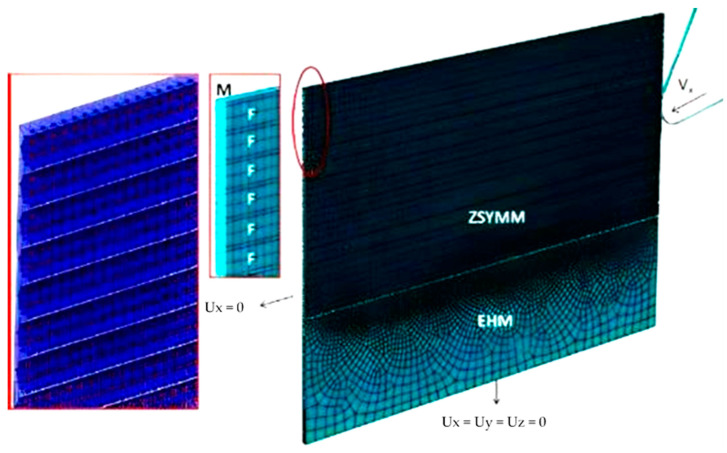
The 3D hybrid model with the applied BCs at the fibre orientation of 0°.

To incorporate the hybrid approach, a 3D model was built and analysed for two fibre orientations of 0° and 90°. A meso-scale technique was employed to simulate the composite material, assuming symmetry to reduce computational time. Figure 2 illustrates a schematic diagram of the FEM-SPH model with the boundary conditions.

The current study used a cutting tool with a larger radius (20 µm) than that used in previous research (5 µm) [48]. To simulate the wider affected section due to the larger cutting radius, a balance between computational cost and accuracy must be considered. For example, simulating cutting with a 5 µm tool radius and a 15 µm depth of cut at a 0° fibre orientation only required three fibres, whereas at least 30 fibres are needed to be considered when using a 20 µm cutting tool and a 100 µm depth of cut, resulting in a significant increase in computational cost. To reduce the computational cost, the meshing size was increased, and the amount of elements was decreased. The mesh size was set at two micrometres along the fibre direction, while the element size in the plane perpendicular to the fibre axis varied depending on the part’s geometry. An SPH particle was added at the centre of each finite element. Tie constraints were imposed between the FE elements and the SPH particles to ensure that the particles followed the elements before material failure. Once a finite element failed and was removed, the corresponding particle’s motion was determined by the adopted constitutive model. Moreover, tie constraints were utilized in the FEM to connect the elements of the cohesive to both the matrix and fibres, and in the homogenization phase, they were connected to the matrix.

To ensure that the SPH particles remain within the simulated strip in the z-direction, lateral surfaces were introduced and a frictionless contact was established between the particles and the rigid surfaces. A friction coefficient of 0.3 was utilized for the interaction between FE, the SPH particles, and the cutting tool. The speed of the cutting tool was adjusted at the maximum experimental speed of 1100 mm/min to reduce computational costs for a given cutting length. For 0° fibre orientation, a cutting tool with a rake angle of 30°, 100 µm cutting depth, and 200 µm cutting length was employed, while for θ = 90°, a cutting tool with a rake angle of 10° and 50 µm cutting depth was utilized. Initially, a classic FEM approach was used to model the cutting process, with a cohesive model implemented between the matrix and the fibre to simulate their interface. If the failure condition occurs during cutting, the connected finite elements convert into SPH particles, and the elements of the cohesive model are regarded as failed. During the simulation, both FE and SPH particles coexist, and the material properties of the particles must be modified when the corresponding FE fails and is deleted. To address this issue, a subroutine algorithm was developed that takes into consideration the constitutive behaviour of the FE, the SPH particles, and the elements of the cohesive model concurrently. The SPH particles can change from being inactive where the particles do not affect the model to being active where they act for the fragmented material.

#### 2.2.1. The FEM-SPH Hybrid Subroutine

**Figure 3:**
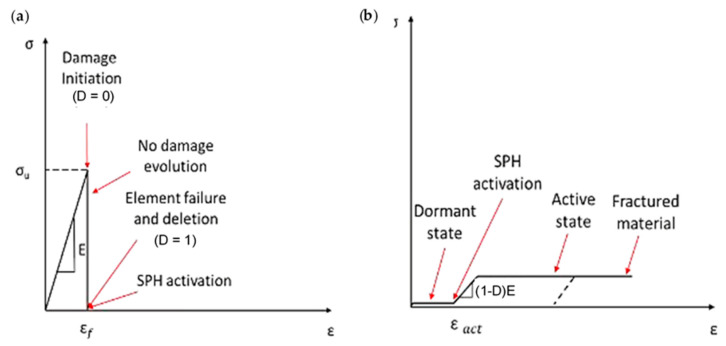
The subroutine incorporates a matrix material model for both (**a**) finite element modelling and (**b**) SPH particles.

An FEM-SPH model consisting of multiple parts was developed, and a subroutine was created to handle the process. The first part involves computing the coordinates of each finite element’s centre and generating SPH particles, ensuring that each FE has an SPH at its centre. The second part links material property models describing the composite material’s constitutive behaviour. The FEM model represents the material from the beginning of the simulation to failure, while the SPH model represents the post-failure behaviour. The VUMAT and VUSDFLD subroutines are employed to compute the connectivity matrices and apply the failure criteria. When a finite element fails, the corresponding SPH changes from dormant to active, and the particle’s stiffness properties are set to a low value to avoid affecting the simulation. However, the material properties are assigned to the SPH if elements fail, and the stiffness degradation is performed to simulate the fractured elements (see Figure 3a). The matrix constitutive behaviour used in the FEM-SPH model differs from the literature models, and a cutting speed of 18.33 mm/s was employed, causing a strain rate increase that affects the matrix stress–strain curve. The matrix was modelled as perfectly elastic until the occurrence of failure, and the fractured material’s constitutive behaviour was employed in the VUMAT subroutine (see Figure 3b). The degradation of material properties is conducted to simulate the behaviour of fractured material. For the matrix material, damage occurs when it reaches a critical stress value called σu. When damage initiates (D = 0), the element fails and is immediately deleted, with no further damage evolution during the finite element (FE) regime. This is depicted in Figure 3b. At this stage, the smoothed particle hydrodynamic (SPH) method takes over, and the deleted elements are replaced with SPH particles. The damage then continues to evolve during the SPH regime until the material fractures completely, which marks the termination of the material. There is no transitional phase between the FE and the SPH regimes.

**Figure 4:**
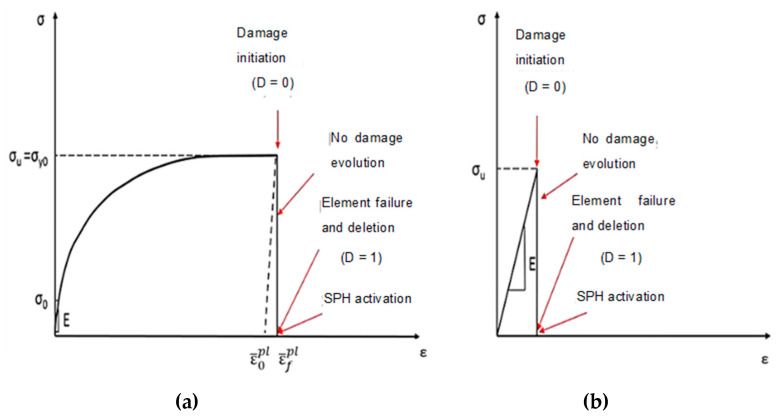
Fibre material model implemented in the subroutine for finite elements (**a**) tension, (**b**) compression.

Compared with the epoxy matrix, carbon fibres exhibit different behaviour under varying rates of strain [27,32]. Figure 4 depicts simulations that were performed to investigate the behaviour of carbon fibres under both tension and compression. The fibres were modelled with transverse isotropy and ideal elasticity up to the point of tensile failure. The criterion for failure was determined by the maximum principal stress. In previous work, Zhou et al. conducted longitudinal compression tests on a single carbon fibre T800S [28], which revealed that the typical stress–strain curve under compression shows elastic–plastic performance. The investigation revealed that the strain at which compression failure occurs was above 10%, indicating a substantially larger value than the brittle failure strain of 2% observed under tension. Additionally, the ratio between tensile and compressive strengths was approximately 0.5, indicating a lower compression stress failure. A Weibull analysis was performed to assess the compression and tensile strength variability, indicating that the compressive strength showed greater variability compared with the tensile strength.

To ensure the reliability and accuracy of the implementation of the fibre behaviour under compression in the hybrid model, the data collected by Zhou et al. [28] was utilized. The compression stress failure was determined based on the strength under tension, and the elasto-plastic behaviour was taken into account until failure occurred. To implement the transversely isotropic behaviour of the fibres in Abaqus/CAE, the Hill criterion was utilized. Once the failure condition was met, whether in tension or compression, the finite element was eliminated from the analysis, and the corresponding SPH particle was activated.

Two simulations were carried out to explore the impact of the value of the damage variable of the matrix (0.1 and 0.8) on the results. In both simulations, the damage variable of the fibre was maintained constant at a value of 0.8 owing to the elasto-plastic properties of fibres under compression and the brittle failure of the matrix material. The majority of damage occurs in the matrix, resulting in a significant number of activated particles, whereas only a few finite elements of the fibres are deleted. Thus, changing the damage variables for fibre particles is unlikely to have an effect on the results, unlike for the matrix particles. Table 1 summarises the material properties of the hybrid model. Abaqus/CAE provides automatic FEM-SPH conversion but the contact between the matrix and the fibre particles must be established after conversion. Using the same material card is the only way to achieve contact, which is possible with the VUMAT subroutine for post-failure behaviour, as in the previously developed 3D-SPH model.

#### 2.2.2. SPH Particle Dimensions

To apply the SPH approach, it is essential to allocate a specific radius to all of the particles. However, defining the particle radius with cylindrical fibres presents a challenge due to the geometry complexity. In the limited gap between two adjacent fibres, the matrix becomes significantly small, requiring a very small particle radius. However, reducing the particle size increases computational time as the SPHs for the model are small. Furthermore, the FE deformation during cutting, particularly in parts subjected to compression stresses, adds to the complexity of choosing the particle radius. Therefore, the SPHs are constrained by a tie to follow the FE. If two neighbouring components undergo compression, they will undergo a deformation and cause a decrease in the distance between their respective particles. Therefore, for optimal outcomes, it is essential to ensure that the particle diameter is smaller than the minimum element in the simulation.

**Figure 5:**
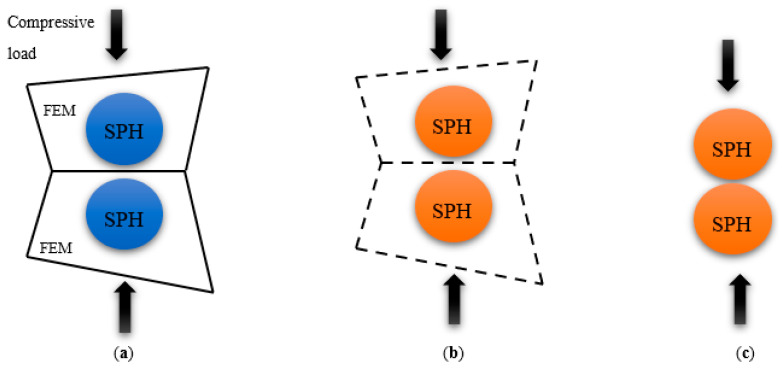
The position and deformation of particles under compression loading in (**a**) dormant, (**b**) activated SPHs, and (**c**) SPH deformation.

Using a uniform radius for SPH particles has two main limitations. First, when a finite element fails, the corresponding SPH particle that becomes active deletes less material during the analysis than what is replaced. Second, thrust force prediction is affected when two FE are removed under the cutting tool because the two activated particles are typically not in contact with each other. To address these issues, a particle radius of 0.7 µm was used, as shown in Figure 5. This smaller particle size allows for more accurate material deletion and compacted fractured material under the cutting tool, leading to a more precise thrust force prediction compared with using SPH modelling alone.

### 2.3. Experimental

**Figure 6:**
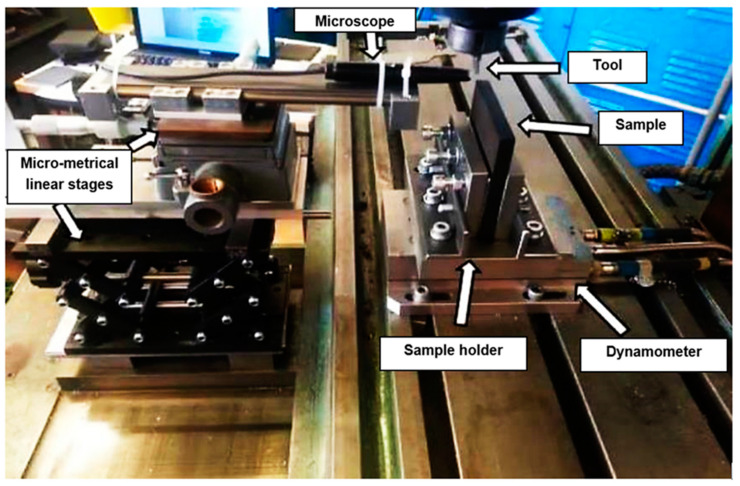
Experimental set-up.

Several studies have used a milling machine in which the tool spindle is locked and the sample is translated with the table [50,51]. For this investigation, Cincinnati milling was employed for machining, with a stationary tool holder and the workpiece moving along a translational path, as shown in Figure 6. To measure cutting and thrust forces during machining, a dynamometer (Kistler tri-axial) was used [51,52,53]. To vertically place the samples on the Kistler dynamometer, a custom-designed holder was created, as described in Kahwash et al. [54] (Figure 6). To study chip types and formation mechanisms, a Supereyes@ digital microscope (Guangzhou, China) was employed to capture photos during the cutting at a rate of 15 f/s. The microscope was placed on the side of the sample facing the area of contact between the cutting tool tip and the sample. It was mounted on three linear stages and equipped with LED. It was also connected to a computer to capture images. Data were collected for the smallest cutting speed within the considered range (12 mm/min) for various fibre orientations, cutting depths, and rake angles. The T800S/HexPly^®^ M21 carbon fibre/resin matrix composite material, consisting of pre-impregnated sheets with a thickness of 0.26 mm matching the fibre diameter of 7.5 microns and a fibre volume percentage of 60%, was prepared; several steps were implemented to enhance the process [55,56]. Initially, the pre-impregnated carbon fibre was meticulously cut into the desired dimensions using a sharp cutter, while ensuring a pristine and uncontaminated work surface through thorough cleaning. Subsequently, a plywood sheet was carefully positioned on the processing table and shielded with release film, followed by the layering of breather fabric and T800 pre-impregnated carbon fibre. To maintain uniformity in the fibre alignment, pen markings were used to indicate the orientation of the fibres, repeating this procedure until the desired number of layers was achieved. Finally, the fabrication of laminates was accomplished, with each layer set to specific fibre orientations of 0°, 45°, 90°, and 135°, respectively.

## 3. Results and Discussion

This section provides the results of the hybrid FEM/SPH model and the FEM model for fibre angles of 0° and 90° which were also compared with the experimental findings. This evaluation aimed to determine the feasibility of using the hybrid approach compared with the commonly used finite element model in simulating machining processes. The hybrid model combines the smoothed particle hydrodynamics approach with micro-mechanical modelling using cylindrical fibres.

### 3.1. Damage and Chip Formation

#### 3.1.1. FEM for 0° Finite Fibre Orientation

**Figure 7:**
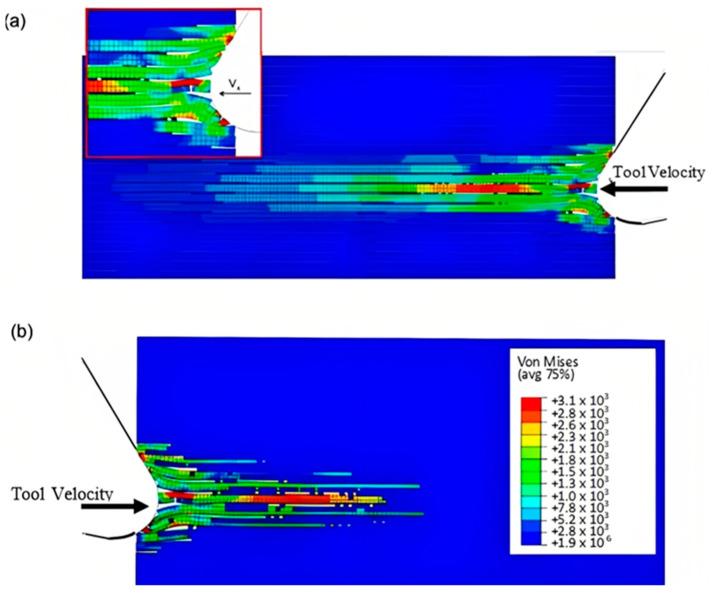
FEM configuration at 5.77 × 10^−4^ s for the 0° fibre orientation (**a**) fibre section, (**b**) matrix section.

The finite element model in Figure 7 depicts the configuration 5.77 × 10^−4^ s after the start of cutting. The tool’s advancement compresses fibres at the tip, causing the central fibre to buckle under axial force. Adjacent fibres bend and deflect on the rake face due to the opening force applied by the tool. Stress propagation extends beyond the tool, leading to brittle failure in the matrix around the tool and along the fibre direction due to significant fibre deformation. This effect is evident in the buckling of the central fibre.

**Figure 8:**
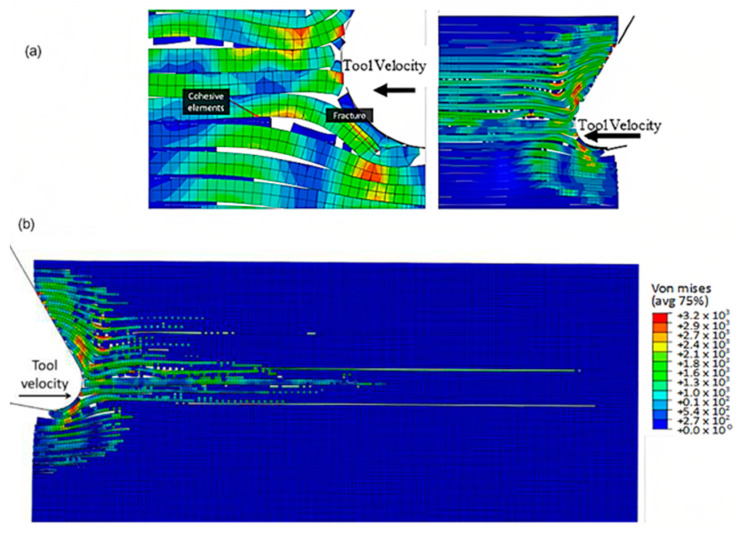
FEM configuration at 1.79 × 10^−3^ s for the 0° fibre orientation, (**a**) fibre section, (**b**) matrix section).

As the cutting process progresses, the fibres located near the tool tip undergo upward bending at a time of 1.79 × 10^−3^ s, leading to fibre failure and the propagation of cracks perpendicular to the fibres’ axis, as depicted in Figure 8. The degree of bending deformation gradually reduces as the fibre located on the sample’s free surface is approached. When buckling occurs due to the compressive load, the central fibres break into pieces and get trapped between the tool and the workpiece. The development of axial forces often causes instability in front of the cutting tool, which may result in buckling failure. As the tool makes contact with the material, the fibres that deflect downwards experience compression, causing the upper section of the fibre in contact with the tool to fracture. This leads to the deformation of cohesive elements that bind the matrix and fibre together. The failure of the matrix then spreads radially outwards from the tool, primarily propagating in front of it in the direction of the fibres until reaching the model boundary.

**Figure 9:**
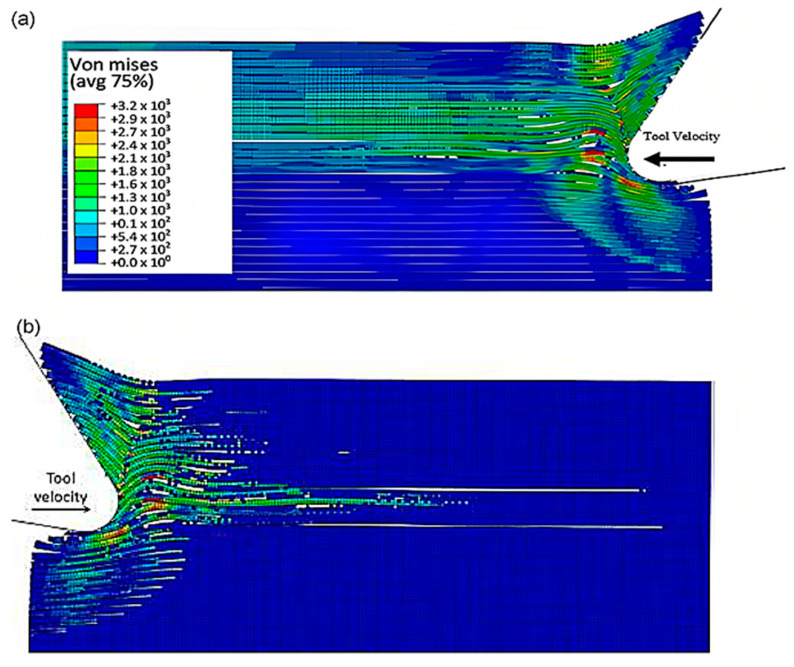
FEM configuration at time 2.84 × 10^−3^ s for the 0° fibre orientation, (**a**) fibre section, (**b**) matrix section.

The model’s configuration at 2.84 × 10^−3^ s is depicted in Figure 9. As the tool moves forward, the fibres above the cutting edge undergo a considerable bending deformation, resulting in the propagation of cracks towards the sample’s free surface through adjacent fibres. An axial load in front of the cutting tool results in an increase in deformation, which damages the fibres located under the cutting plane. This damage is visible in the form of cracks caused by compression. Furthermore, there is a significant rise in the matrix fracture, mainly near the tool, where most of the matrix elements are eliminated as they fail.

**Figure 10:**
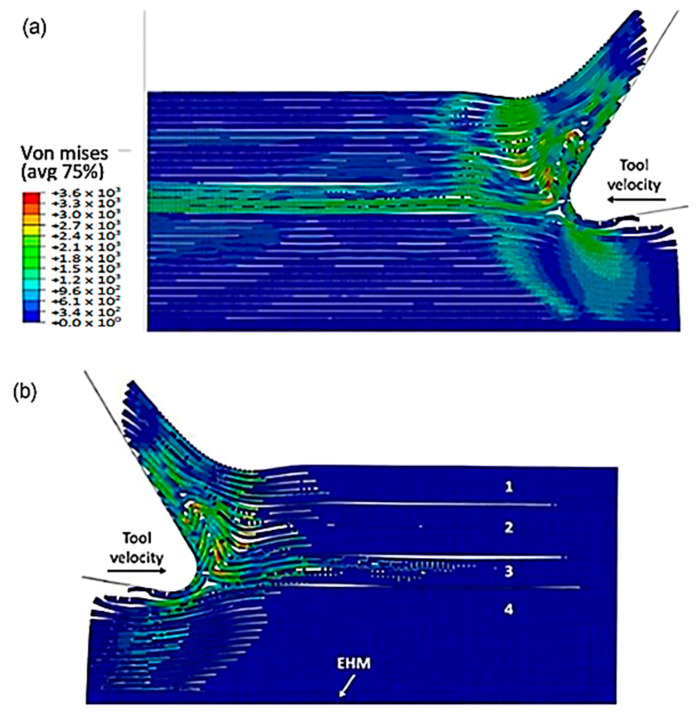
FEM configuration at 4.5 × 10^−3^ s for the 0° fibre orientation, (**a**) fibre section, (**b**) matrix section.

As the tool advances further, at a time of 4.5 × 10^−3^ s, the fibre’s buckling instability leads to the failure of fibres ahead of the tool and results in multiple fractures, as shown in Figure 10. The cutting area expands, causing the material to bend and slide along the tool’s rake face, while the compression zone below the tool also widens, and the matrix fracture may even extend to the EHM phase. To gain an understanding of the matrix failure within the material, a more comprehensive model is required. Based on the material deformation, the sample can be split into four horizontal strips. Strips one and four exhibit primarily fibre bending behaviour, while strips two and three experience mostly buckling. However, the fibre behaviour tends to shift towards bending as we move towards strips one and four.

**Figure 11:**
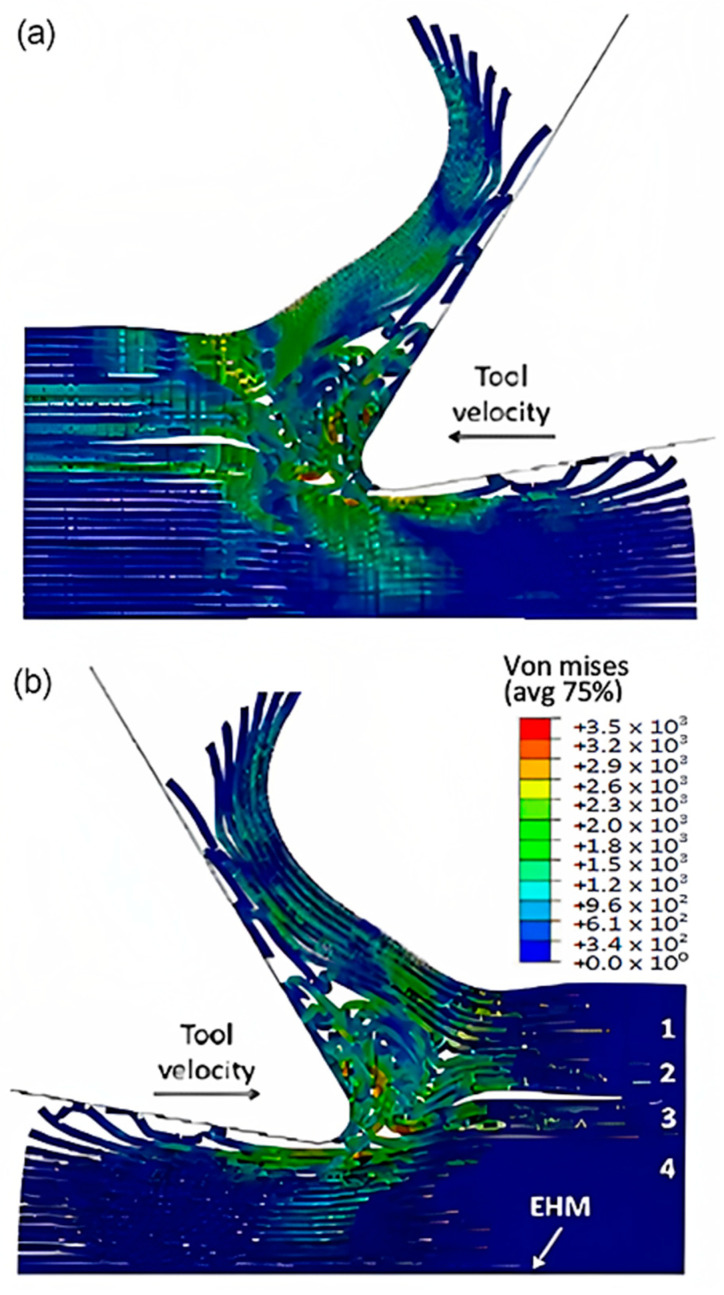
FEM configuration at 1.09 × 10^−2^ s for the 0° fibre orientation, (**a**) fibre section, (**b**) matrix section.

Figure 11 depicts the model configuration at the end of the simulation, which still highlights four distinct strips. Of particular note are strips two and three, which are particularly susceptible to buckling-induced fibre failure. As the tool advances, the broken fibres accumulate ahead of it, forming a cluster that the tool uses to exert an opening force on the material. Strip one bends and deforms on the cluster ahead of the cutting tool, while strip four’s fibres begin to deform downwards in advance of the tool. The damage to the matrix is extensive and may even extend beyond the cutting plane, creating a formidable challenge for the tool’s lifespan. Ultimately, the process produces a continuous chip that glides onto the rake face of the tool, which worsens the wear and deterioration of the tool as time passes.

**Figure 12:**
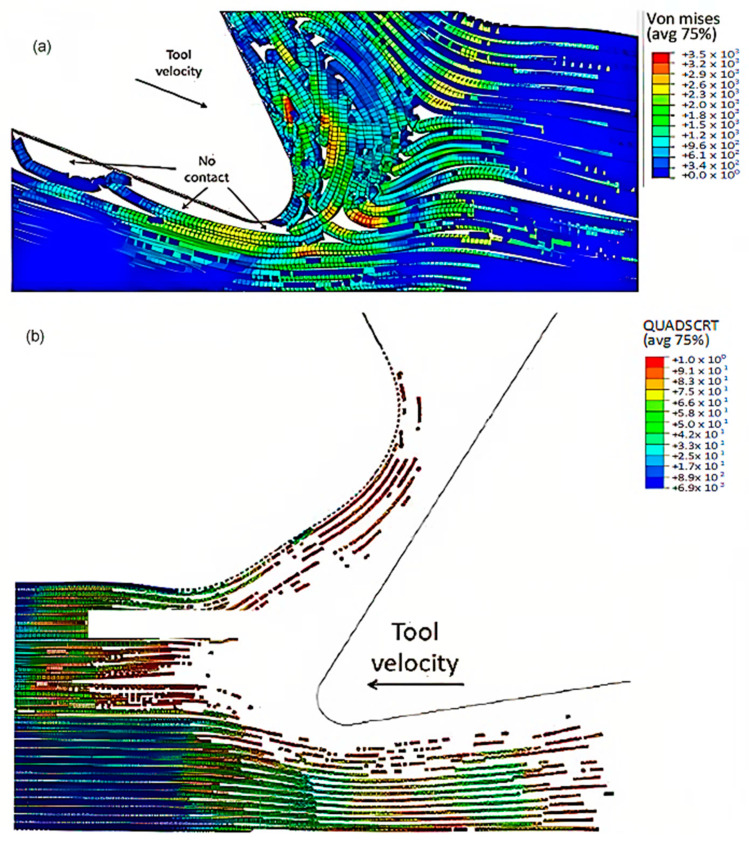
(**a**) Tool-tip workpiece, (**b**) cohesive elements configuration.

To accurately analyse element failure, it is crucial to have a clear understanding of the workpiece and limited contact of the clearance face. This phenomenon can have significant implications on the overall process, so it must be carefully observed and acknowledged, as shown in Figure 12a. The configuration of cohesive elements after the analysis is displayed in Figure 12b, and the QUADSCRT variable provides crucial information about the damage initiation condition and which cohesive elements are damaged but have not failed yet. Two primary factors contribute to the clumping of fibres in front of the cutting tool, ultimately leading to the failure of cohesive elements. Firstly, a matrix failure causes the deletion of linked cohesive elements. Secondly, extensive deformation of fibres causes cohesive elements to stretch until they break, resulting in debonding between the matrix and fibre. Cohesive FE elements located near the fibre agglomeration are more likely to fail as the tool advances. Therefore, appropriate measures must be taken to prevent any potential problems. It is essential to understand that the breakdown of cohesive components in the compressed area under the cutting plane is strongly connected to the collapse of the matrix and must be considered during the analysis. Thus, understanding this phenomenon is critical to optimize the process and ensure a successful outcome.

#### 3.1.2. FEM-SPH Model for 0° Fibre Orientation

a At damage variable (D) of =0.8.

**Figure 13:**
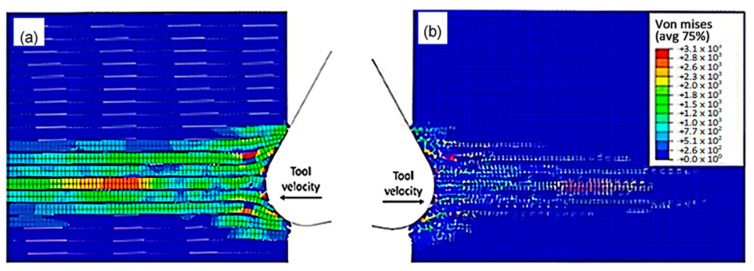
FEM-SPH hybrid model configuration at 5.77 × 10^−4^ s for the 0° fibre orientation and D = 0.8, (**a**) fibre section, (**b**) matrix section.

The SPH matrix system was subjected to a 0.8 damage variable and the hybrid model was implemented to obtain results, and these were compared with the classic FEM using the same time steps. The model’s configuration at 5.77 × 10^−4^ s was illustrated in Figure 13. It was observed that the finite elements in the FEM-SPH model exhibited a behaviour similar to the classic FEM results. The central fibre was found to buckle, and the surrounding fibres were subjected to bending because of the cutting opening action. The key difference between the two models, however, was the fact that the finite elements that were removed from the hybrid model were transformed into particles.

**Figure 14:**
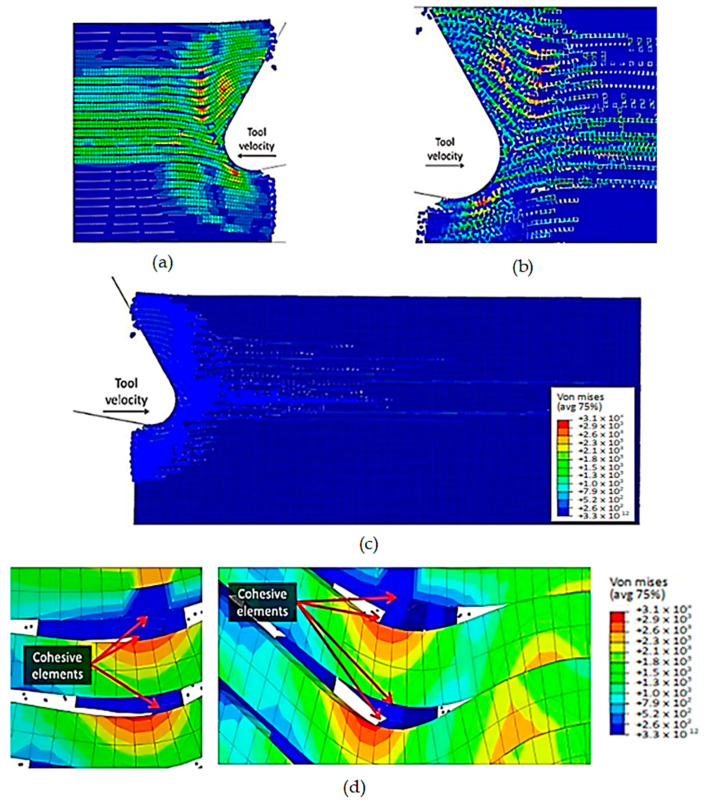
FEM-SPH model at 1.79 × 10^−3^ s for the 0° fibre orientation and D = 0.8, (**a**) fibre section, (**b**) magnified matrix section, (**c**) matrix section, (**d**) cohesive elements behaviour.

The brittleness of the matrix makes a great impact on the cutting process. At 1.79 × 10^−3^ s (as depicted in Figure 14), there is an increase in the bending of the fibres located above the cutting edge, ultimately leading to failure. Meanwhile, the fibres located below the machining plane bend downwards, causing cracks to appear in the material. This is the point at which the differences between the FEM and hybrid models become apparent. When observing the hybrid model, two notable features can be identified. Firstly, there is a noticeable build-up of particles around the tool edge. Secondly, due to the damaged material, a larger amount of the material is influenced during cutting. Consequently, stress propagation is significantly more extensive in the hybrid model when compared with the FEM. This is noticeable in the workpiece upper corner, where the hybrid model shows upward bending deformation, whereas the FEM depicts the fibre remaining in its original configuration (as shown in Figure 14a–c). Furthermore, the finite elements representing the matrix material in the corner fail in the hybrid model, which is different from the FEM. Hence, the impact of the tool’s opening action is not limited to the damaged material but also extends to the material located at a significant distance from it. The FEM model indicates that the compression of fibres causes the stretching of cohesive elements, leading to debonding in the area ahead of the cutting tool, as shown in Figure 14d. Despite this, a significant number of cohesive elements were eliminated from the model due to the matrix failure, highlighting the material’s brittle behaviour.

**Figure 15:**
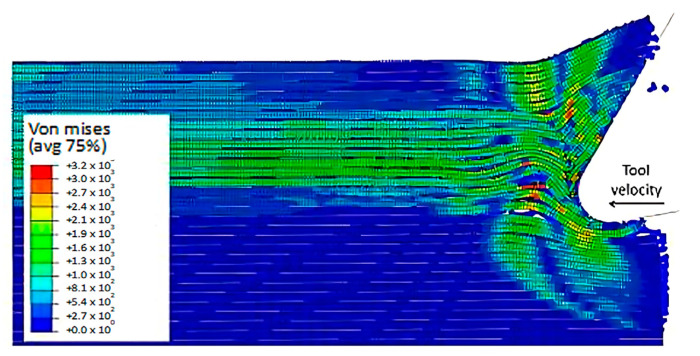
Hybrid model configuration at 2.85 × 10^−3^ s for the 0° fibre orientation and a matrix damage variable of 0.8.

The simulation results show that both the FEM and the FEM-SPH approaches are similar when the time step is 2.845 × 10^−3^ s, as shown in Figure 15. During the cutting process, a crack is observed to travel perpendicularly from the tool’s cutting edge towards the workpiece surface. This behaviour is caused by several failure mechanisms. The crack initiation occurs in front of the tool due to buckling, and then it expands away from the edge because of bending failure. The model also demonstrates that the material under the cutting tool undergoes compression, leading to visible deformations of the particles at the sample’s vertical free edge, which protrude outward.

**Figure 16:**
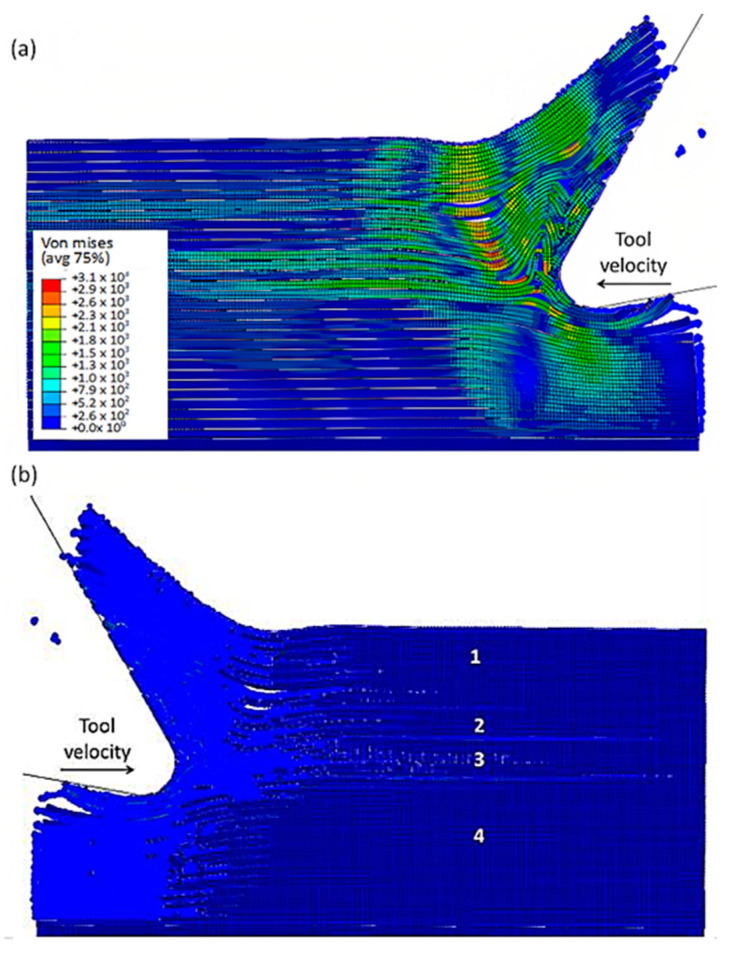
FEM-SPH model configuration at 2.85 × 10^−3^ s for the 0° fibre orientation and a matrix damage of 0.8, (**a**) fibre section, (**b**) matrix section.

At 4.527 × 10^−3^ s, as shown in Figure 16, the hybrid model demonstrates a similar agglomeration of fractured fibres and the matrix as the FEM. However, in contrast to the FEM model, the failed matrix elements in the agglomeration were clearly shown in the hybrid model. The area located in front of the tool can be categorized into four separate strips, with strips one and four exhibiting bending of fibres and strips two and three showing primarily buckling. Additionally, the tool’s movement causes the SPH particles to accumulate around the cutting edge.

**Figure 17:**
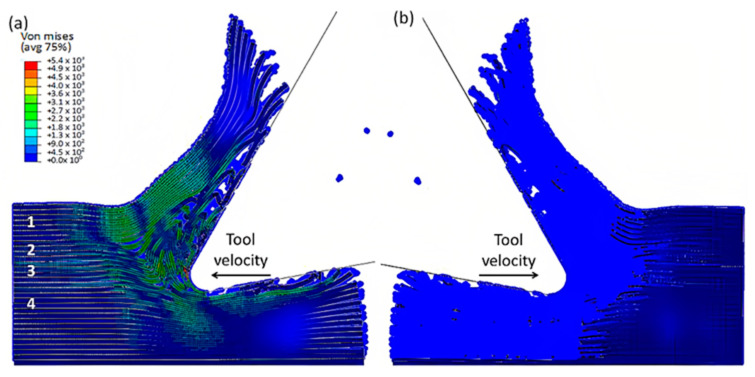
FEM-SPH model at 2.85 × 10^−3^ s for the 0° fibre orientation and a matrix damage of 0.8, (**a**) fibre side, (**b**) matrix side.

The comparison of the two models is more evident in Figure 17 when the time step is 1.09 × 10^−2^ s. Even though both models generate continuous chips, the hybrid model produces a larger chip than the FEM because there is more material near the cutting edge that prevents the tool from losing contact with the workpiece and avoids separation. The thrust force that occurs is caused by the pressure from the material acting on the clearance face. Additionally, the SPH model has the capability to predict and simulate the bounce-back phenomenon. When the material particles fail, they interact with the remaining SPH particles based on the assigned constitutive model, creating a cracked material that can undergo elastic recovery.

**Figure 18:**
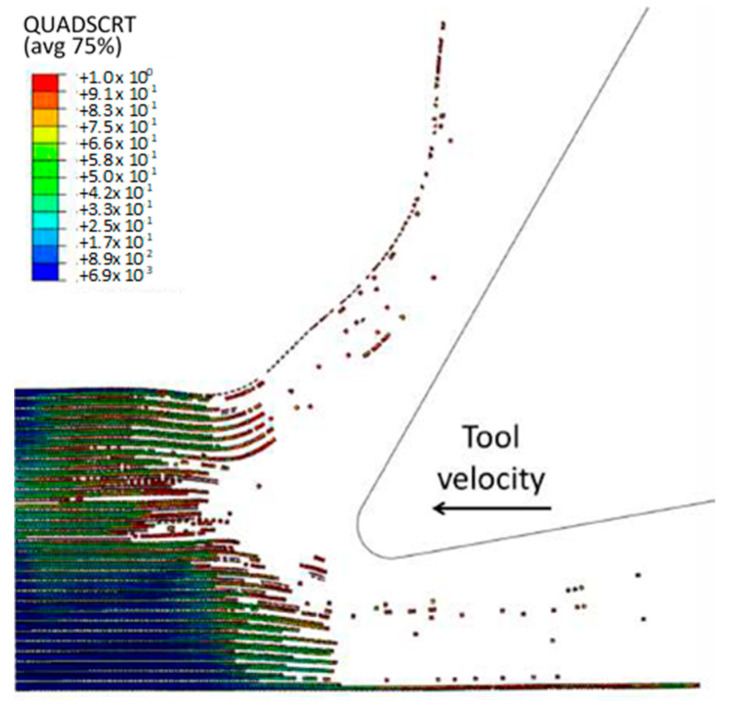
The condition of the cohesive elements after the simulation is completed in the FEM-SPH model for the 0° fibre orientation using a matrix damage of 0.8.

The comparison between the FEM-SPH hybrid model and the finite element is demonstrated in Figure 12b and Figure 18. The latter figure shows that a greater number of cohesive elements have failed in the hybrid model, which indicates more damage in the matrix phase due to matrix failure under the tool. The wider extent of the failure of the cohesive element in the hybrid model is likely due to the presence of damaged material in the model. This results in a larger volume of material being involved in the cutting process, in all directions. As a result, more cohesive elements fail in the hybrid model due to matrix failure, as compared with the finite element model.

b At damage variable (D) of 0.1.

**Figure 19:**
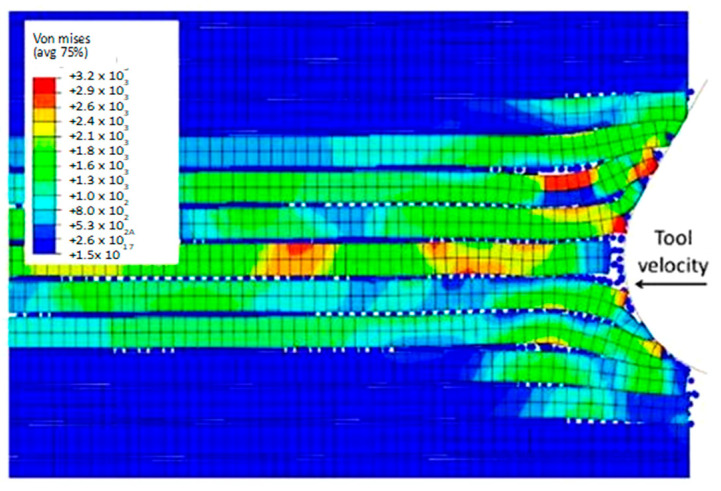
Hybrid model at 5.77 × 10^−4^ s for the 0° fibre orientation using a matrix damage of 0.1.

In the current simulation, a damage variable (D) of 0.1 is utilized at a specific time step of 5.77 × 10^−4^. Figure 19 shows a slight deviation from the previous models, where the fibres at the centre fail near the advancing tool, leading to their compression and bending. The presence of stiffer, broken material in front of the cutting tool triggers local failure due to compression instability before buckling, which was observed in the earlier models. This phenomenon results in a deviation from the previous models and highlights the importance of the material’s stiffness and the impact of its failure on the cutting process.

**Figure 20:**
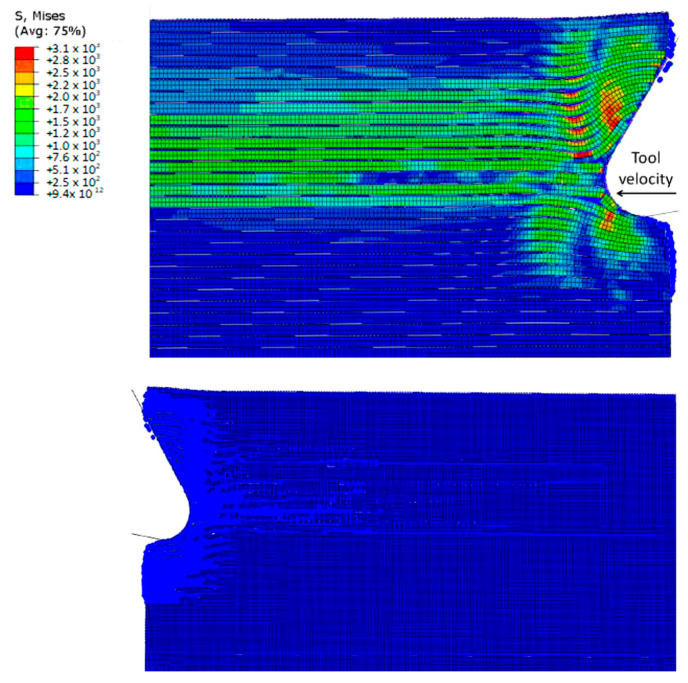
FEM-SPH model configuration at 1.79 × 10^−3^ s for the 0° fibre orientation and D = 0.1, (**a**) fibre section, (**b**) matrix section.

At a time of 1.79 × 10^−3^ s (as depicted in Figure 20), the previously mentioned phenomenon becomes more apparent. Specifically, during the cutting process, the finite elements of the matrix that are located near the fibres begin to fail. When a stiffer and broken material is introduced into the matrix, it can decrease the deformation of the fibres caused by axial loads, leading to their failure due to compression stress. This occurrence enables us to observe how the tool motion affects the sample, causing the fibres to bend upwards above the cutting edge and downwards below it. Consequently, as a result of the downward force exerted by the tool, fractures and cracks emerge in the fibres located below it.

**Figure 21:**
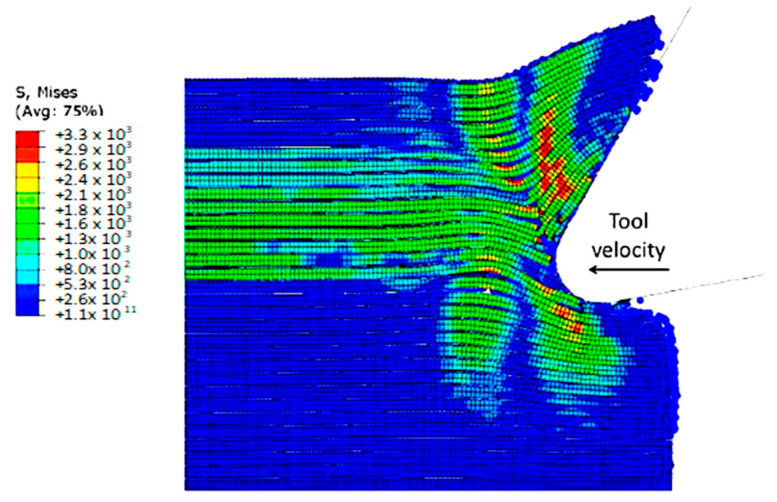
Hybrid model configuration at 2.85 × 10^−3^ s for the 0° fibre orientation using a matrix damage variable of 0.1.

As the cutting tool moves forward, the fibres located in front of the cutting area start to undergo deformation as a result of axial forces, as depicted in Figure 21. Although the degree of these deformations is relatively lower than that observed in earlier simulation models, they still occur. Moreover, a crack is observed to propagate through the nearby fibres in the direction of the sample’s free edge, but its length is comparatively shorter than that in previous models. The axial load deformations in the fibres continue to increase as the tool progresses, ultimately leading to buckling instability and failure.

**Figure 22:**
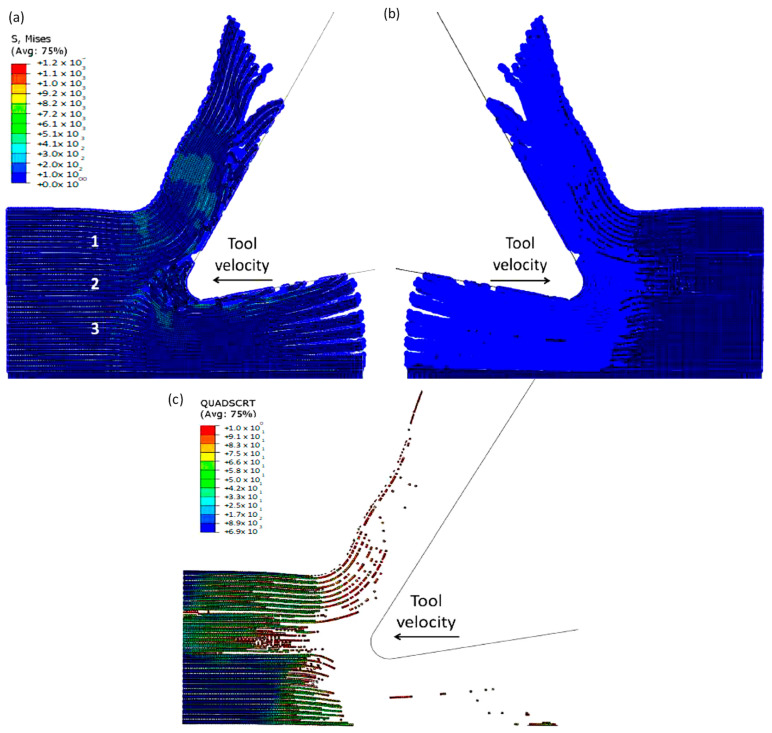
(**a**,**b**) Hybrid model configuration at 1.09 × 10^−2^ s for the 0° fibre orientation when implementing a matrix damage of 0.1, (**c**) cohesive elements’ configuration.

Figure 22a,b exhibit a noticeable contrast when compared with previous models. In particular, only a small portion of fibres in front of the tool experience buckling failure, while most of them undergo bending deformation. This results in the formation of a smaller agglomeration, which remains in close proximity to the tool tip. The study indicates that fractured material has a higher stiffness, enabling it to bounce back more due to a greater volume of material in contact with the clearance face of the cutting tool. Figure 22c displays the final configuration of cohesive elements, which depicts a wider region with failed cohesive elements in comparison with the FEM model shown in Figure 12b. Additionally, there are failed cohesive elements in the formed chip. Due to the failure of the matrix finite elements, most of the cohesive elements experienced failure beneath the cutting plane. Hence, it can be inferred that the damage extends beyond the area of the micro-mechanical model. Therefore, it is recommended to incorporate a greater number of fibres in the model to assess the extent of the damage.

#### 3.1.3. FEM for 90º Fibre Orientation

**Figure 23:**
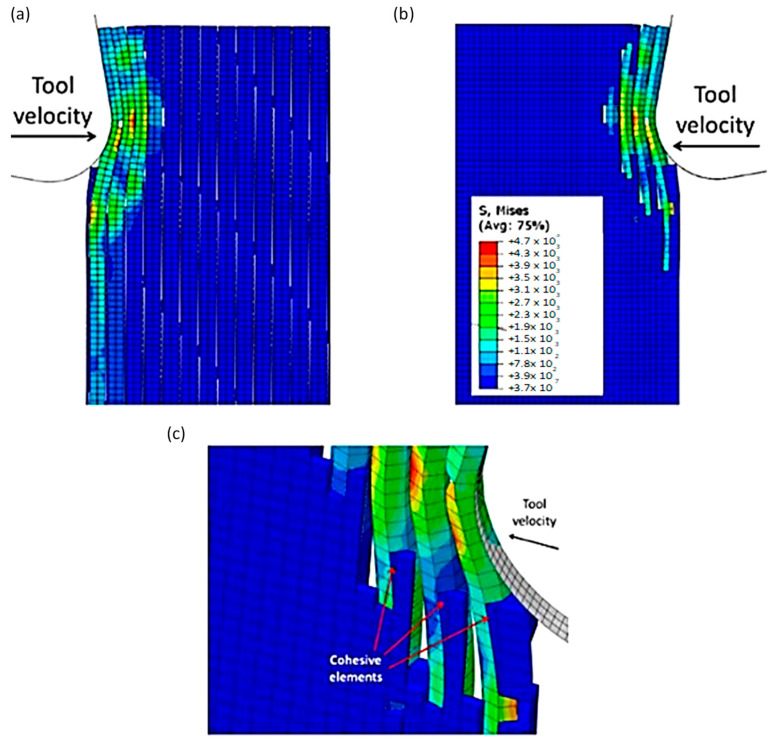
FEM configuration at 4.2 × 10^−4^ s for the 90° fibre orientation, (**a**) fibre section, (**b**) matrix section, (**c**) Cohesive elements behaviour—Matrix side.

The formation of chips in CFRP composites with a fibre orientation of 90º is noticeably different compared with those with a 0º fibre orientation. When the cutting tool is perpendicular to the fibre direction, a unique mechanism for chip formation occurs. At the beginning of the cutting process, a fracture takes place in the matrix, extending in front of the cutting area and beneath the cutting plane, due to the bending of the fibres by the cutting tool (as illustrated in Figure 23). Significant deformation occurs throughout all phases of the material, as demonstrated by the behaviour of cohesive elements in Figure 23c. These elements undergo substantial deformation in order to maintain the connection between the matrix and fibres, leading to debonding and stretching of the cohesive elements during cutting. Once a fibre comes into contact with the tool, it begins to fracture, with the crack propagating along the fibre at locations where the maximum principal stress criteria are met. However, the fibre is not fully severed at this stage.

**Figure 24:**
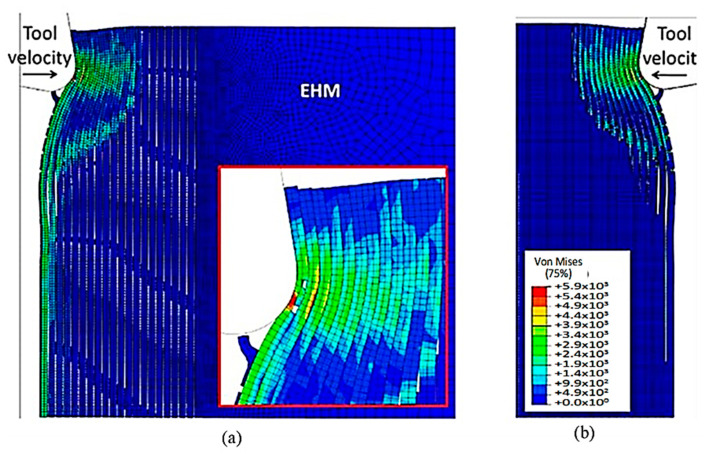
FEM configuration at 1.46 ×10^−3^ s for the 90° fibre orientation (**a**) fibre section, (**b**) matrix section.

Figure 24 shows that as the cutting tool advances, a substantial amount of matrix element failure occurs in areas far ahead of the tool and under its path. This failure causes the removal of matrix elements, leading to contact between adjacent fibres. As the tool continues to move forward, the movement is transmitted through these fibres, resulting in significant bending. The fibre that comes into contact with the cutting edge experiences compression in the cutting direction, which initiates a new crack below the previously formed crack. The fracture in the matrix then propagates along the first fibre, almost reaching the boundary of the model, deep within the workpiece.

**Figure 25:**
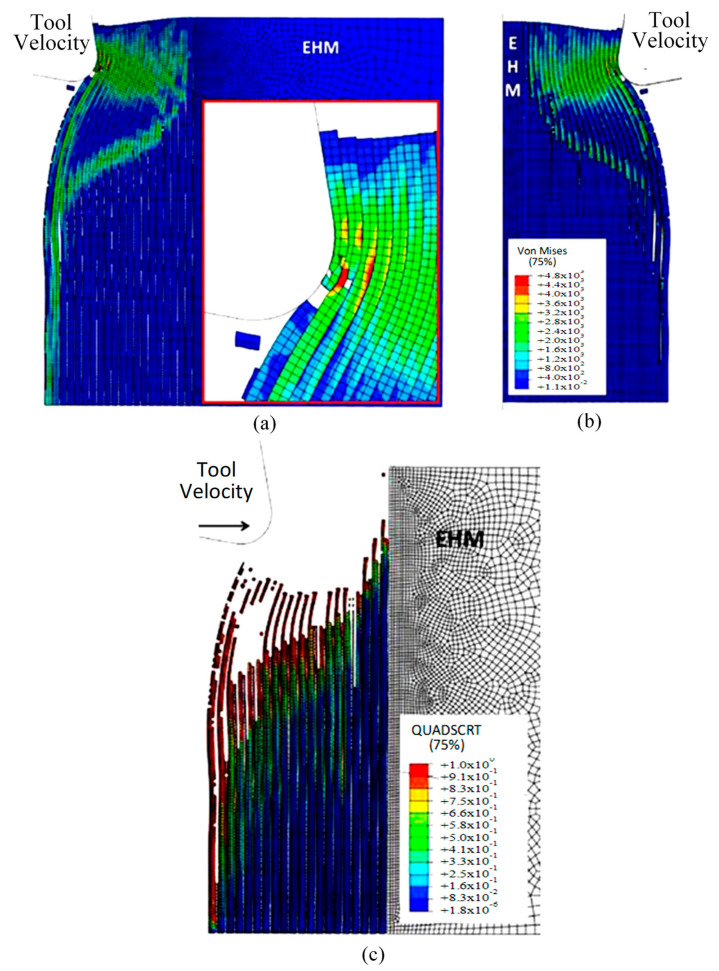
(**a**,**b**) FEM configuration at 2.26 × 10^−3^ s for the 90° fibre orientation, (**c**) cohesive elements’ configuration.

Figure 25 shows that, as the cutting tool moves forward, an increasing number of fibres are involved, and the degree of fibre bending intensifies, as shown in the figure. The first fibre may develop cracks that cause it to split into two parts. The top portion of the sample generates a chip that moves slowly on the tool rake face, whereas the bottom portion of the sample slides towards the open face of the cutting tool. Additionally, cracks also occur in the second fibre. As the fibres bend more, the matrix’s failure extends in front of the cutting tool and deep into the sample. The failure of cohesive elements takes place well ahead of the tool and below the cutting path. Figure 25a–c show that the failure of cohesive elements is primarily due to the deletion of matrix elements. It can also be noted that the elastic recovery of the first fibre, after the passing of the cutting tool, causes bends of the sample in the opposite direction.

#### 3.1.4. FEM-SPH for 90º Finite Orientation

**Figure 26:**
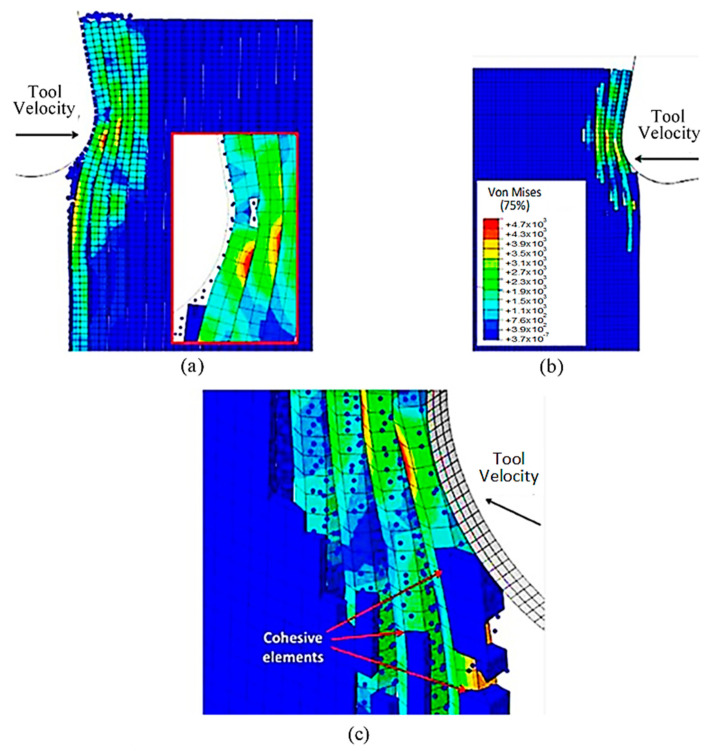
FEM-SPH configuration at 4.2 × 10^−4^ s for the 90° fibre orientation when D = 0.1.

The damage variable value for the SPH matrix was set at D = 0.1. At the start of the analysis, a noticeable difference between the hybrid and FEM models was observed, as shown in Figure 26. As the tool progressed, the fibres bent, resulting in matrix and cohesive elements failure, which had been previously observed. However, the hybrid simulation exhibited a more extensive crack in the first fibre, along with a higher number of fibres bending ahead of the cutting tool than the FEM model. Specifically, the fourth fibre in the hybrid approach started to bend and had a significantly higher Von Mises stress magnitude (2.35 × 10^3^ MPa compared with 1.17 × 10^3^ MPa), resulting in a large area of failed matrix and cohesive elements. The discrepancy in behaviour between the two models was due to the existence of fractured material, which was indicated by the presence of SPH particles. The element deletion typically reduces stress levels by reducing the amount of material. However, the transformation from FEM to SPHs resulted in the fractured elements transferring the cutting tool action to the undamaged workpiece. In the FEM, the cohesive elements experience significant deformation as they try to keep the matrix and fibre phases together.

**Figure 27:**
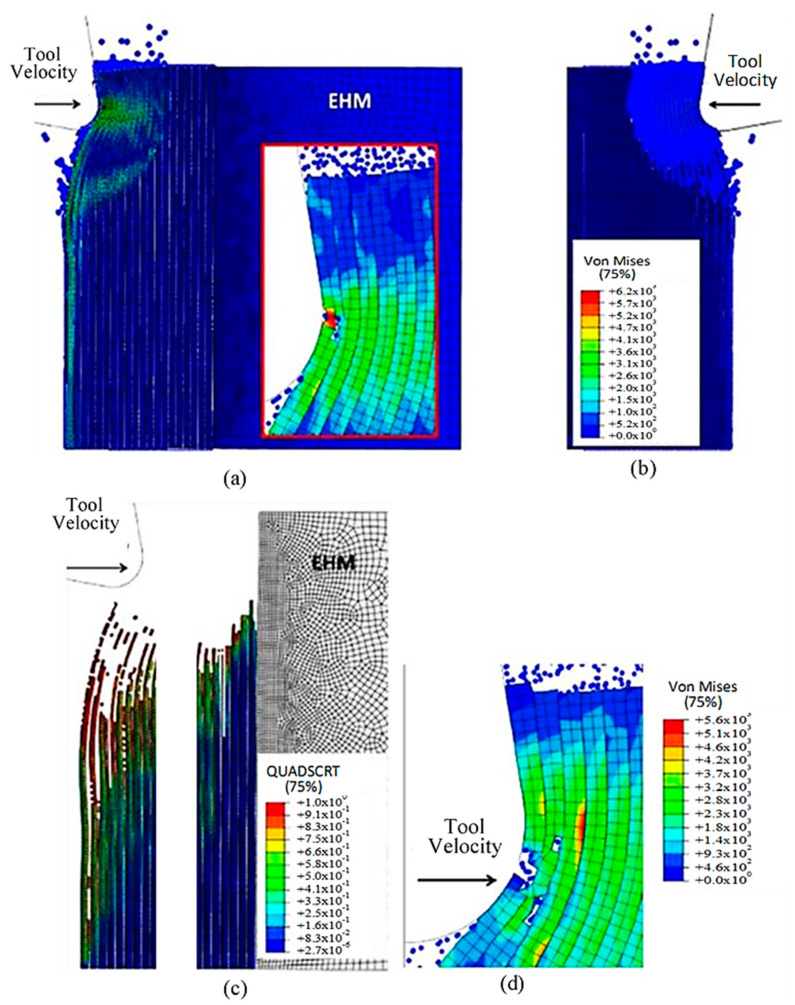
(**a**,**b**) FEM-SPH model at 1.46 × 10^−3^ s, (**c**) cohesive elements at 1.46 × 10^−3^ s, and (**d**) FEM-SPH model at time 2.72 × 10^−3^.

Figure 27a,b demonstrate that as the cutting tool advances, the fibre bends resulting in the relevant matrix elements transforming into SPHs. This transformation from FEM to SPHs enables the model to simulate the formation of a powder chip, where particles scatter from the cutting zone in a powder-like pattern. The primary cause of cohesive element failure is the deletion of matrix finite elements, as shown in Figure 27c. The damage extension in the active cohesive elements extends significantly below the cutting plane, as indicated by the red regions, and many of these elements are on the verge of experiencing damage initiation. In the final analysis, fibre failure reveals the potential path of the developed crack (Figure 27d). The first fibre splits into two parts, with the upper part moving at a slow pace on the rake face, while the second part is damaged multiple times but remains intact. In this case, fibre failure also occurs above the cutting plane, which is consistent with the FEM.

### 3.2. Experimental Validation

To validate the simulation data obtained from both the FEM and FEM-SPH models, machining of CFRP samples were carried out using a milling process. The comparison between the experimental and simulated results includes cutting and thrust forces as well as chip formation.

#### 3.2.1. The Cutting Forces

**Figure 28:**
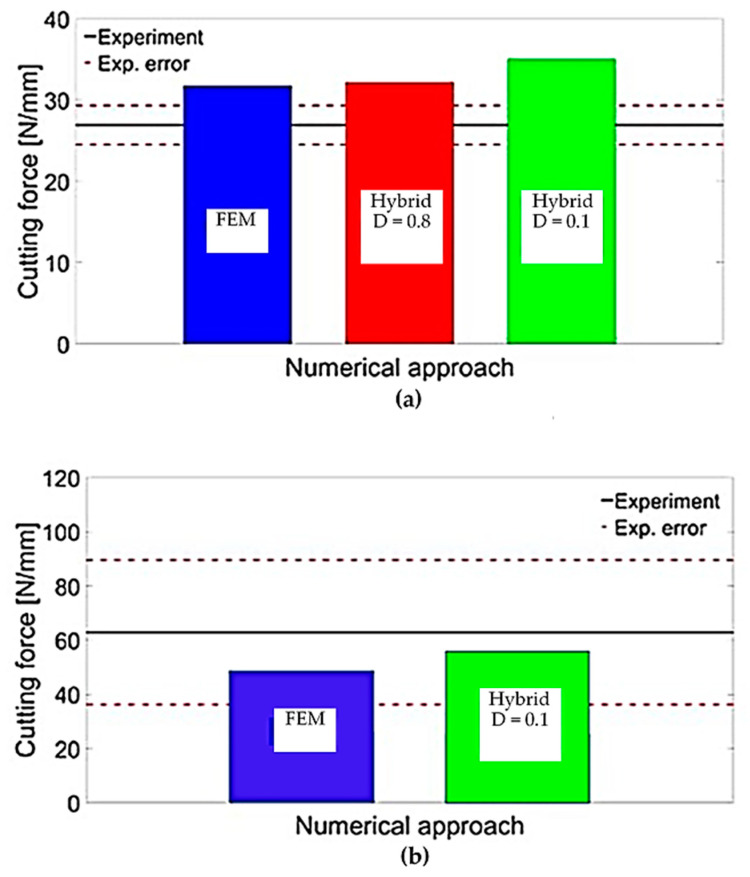
Validation of the cutting forces for fibre orientation of (**a**) 0° and (**b**) 90°.

In Figure 28, the cutting forces obtained from the FEM and hybrid models were compared with experimental values. The results showed that for the 0° fibre orientation, both models predicted similar cutting forces, but the FEM was in better agreement with the experimental. When the FEM-SPH model used the smaller model’s matrix damage variable, the cutting force slightly increased from 32.17 N/mm to 35.05 N/mm. The increase in cutting force was attributed to the conversion of elements to SPHs, where the failed material facilitated the action of the tool to the undamaged parts and compressed the surrounding material. By lowering the damage variable, the damaged material became stiffer, leading to an increase in cutting force. For the 90° fibre orientation, both models predicted cutting forces slightly lower than the experimental value, but the difference was within the margin of the error. The hybrid model’s predicted force exceeded that of the FEM. However, the simulation did not fully replicate the chip formation mechanism because of the high computational cost required to observe its repetition during cutting. As the tool advanced during the simulation, many fibres were involved and bent, which causes a rise in the cutting force and a longer cutting length.

Table 2 summarizes the results of the thrust force obtained from the developed simulations and the experimental results. The table shows that both models underestimated the predicted thrust force, regardless of the fibre orientation. The incomplete simulation of the chip formation mechanism had a negative impact on the accuracy of the thrust force prediction for the 90° fibre orientation. As the simulations approached their end, the fibres were bent ahead of the cutting tool, and the tool made contact with the workpiece at both the cutting edge and rake face. However, there was insufficient tool displacement to allow the machined surface and the clearance face to come into contact, which may have also contributed to the underestimation of the thrust force. In other words, the simulation did not fully account for all the forces acting on the cutting tool. This underestimation may be due to the limitations of the numerical models or the experimental setup. Nevertheless, the results indicate that further improvements are necessary to enhance the precision of the numerical simulations and reduce the discrepancy with the experimental results.

Compared with the hybrid model, the FEM model inaccurately projected the thrust force with significant errors, displaying negative values contrary to experimental observations. The negative thrust force estimation resulted from factors such as fractured fibre accumulation above the cutting plane and the absence of contact between the tool clearance face and the workpiece due to element failure and removal. In contrast, the FEM-SPH (D = 0.8) model provided a more realistic simulation by allowing failed material accumulation and contact with the tool clearance face, resulting in an upward force countering the material’s downward action on the rake face. The FEM-SPH (D = 0.1) model further improved thrust force prediction by simulating stiffer damaged elements accumulating in front of the cutting tool, resulting in an upward force exerted by the workpiece. Overall, the hybrid model accurately predicted thrust force by simulating damaged material and its effects on the cutting forces.

#### 3.2.2. Chip Formation and Type

**Figure 29:**
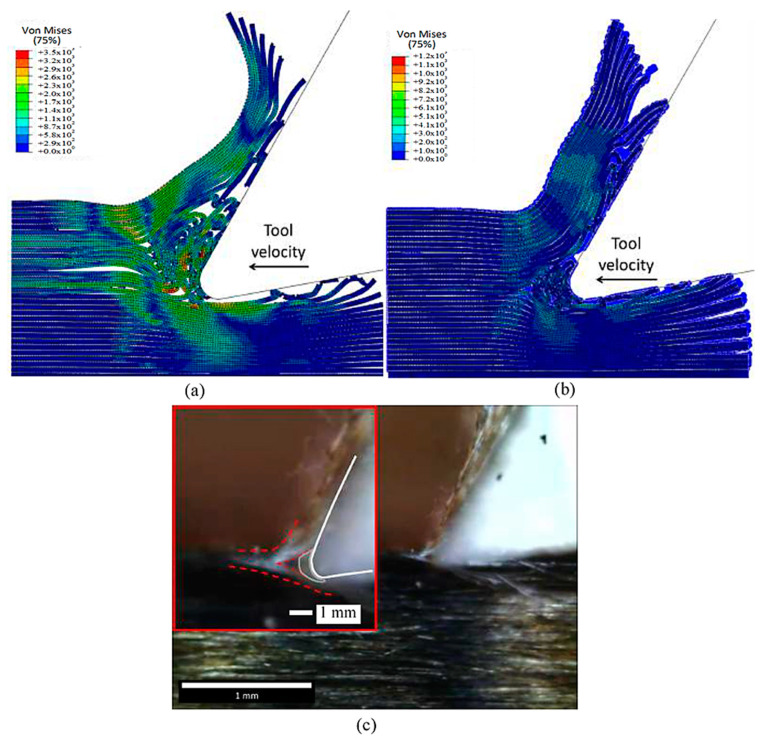
The chip developed during cutting for the fibre orientation at 0° in the (**a**) FEM, (**b**) FEM-SPH model, and (**c**) experimental results.

**Figure 30:**
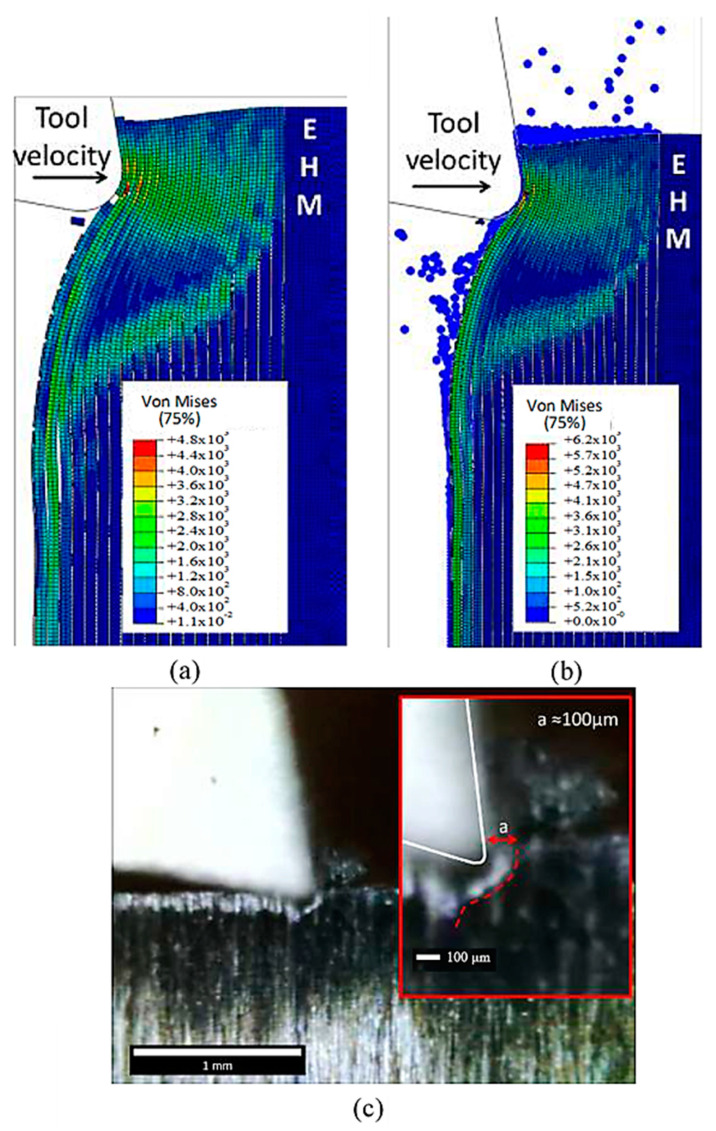
The formed chips for the fibre orientation at 90° in (**a**) FEM, (**b**) FEM-SPH model, and (**c**) experimental results.

Figure 29 and Figure 30 present a comparison of experimentally captured images of chip formation with the developed simulation models. However, due to camera limitations, the captured images were only taken at a low cutting speed and were, therefore, only suitable for qualitative analysis. As the tool moves forward, it compresses the fibres and the matrix along their length, causing damage near the point where the tool meets the material. Unlike the FEM model, which removes elements from the analysis, here the material properties degrade instead. This means that the damaged material gets trapped between the tool and the undamaged part of the material above it. With further tool advancement, the damaged area grows and a crack spreads horizontally along the boundary between the fibres and the matrix. The damaged material helps transfer the tool’s effect to the undamaged areas, allowing the material to open up. During crack propagation, the fibres bend. Once the crack reaches a length of about 30 μm, it suddenly changes direction and moves through the fibres towards the surface of the sample. At the same time, it also moves through the matrix. When the crack reaches the surface, the chip, made up of the cut fibres and the matrix, breaks off, and the process starts again [57,58,59,60,61].

In the case of the 0° fibre orientation, both the FEM-SPH model and classic FEM generated continuous chips, similar to the experimental observations. However, the classic FEM generated a curling chip, while the hybrid model maintained a chip surface that remained almost parallel to the rake face of the cutting tool, which closely resembled the experimental observation. The difference in chip shape is attributed to the fractured material present in the FEM-SPH model, which, although weak, helped to keep the fibres together. Both models demonstrated similar behaviour in workpiece opening, with the upper part of the workpiece bending upwards to form the chip while sliding on the tool rake face, and the lower part of the workpiece bending downwards under the tool.

It is worth noting that the amount of accumulated material in front of the cutting edge observed in the experimental images is similar to that developed in the FEM-SPH approach, although it is a small quantity. Both approaches exhibit fibre bending under the cutting tool, resulting in multi-fractured material, which is consistent with the experimental observations on the machined surface. However, the machined surface in the FEM model shows separated fractured and curled fibres, whereas the fractured material in the FEM-SPH model helps to keep the fibres together. Moreover, the top part of the machined workpiece in the FEM-SPH model is composed of fractured fibres within the damaged material, which is similar to what can be observed in the experimental images.

The experimental observations validate the accurate implementation of the FEM-SPH approach, which maintains the tool’s contact with the material’s clearance. Both approaches also exhibit similar material deformation during cutting, which aligns with the experimental images. The red line marking the material deformation is similar in both models as well. However, the cutting process’s complexity suggests that a larger model may be needed to capture the complete process. Both approaches exhibit damage to the fibres above the cutting surface, where they slide against the rake face, and significant matrix damage is evident, leading to the formation of a chip with a powder-like texture.

## 4. Conclusions

A new hybrid model is proposed to address the limitations of the commonly used FEM method in simulating machining processes. This model combines SPH particles with finite element conversion to overcome the limitations of the classic FEM method. The hybrid model’s performance was compared with the FEM and validated experimentally in a test based on orthogonal cutting of CFRP composites. The results demonstrate that the hybrid model provides a better estimation of thrust force and exhibits better agreement with experimental observations in terms of force direction for CFRP with fibre orientations of 0° and 90°. The FEM-SPH model advantage is attributed to the real presentation of the accumulated damaged particles around the cutting tool, which generates a force acting upward similarly to the experimental work. The differences between the two approaches became more evident when more finite elements failed during the analysis. The FEM-SPH hybrid model’s material removal mechanism is also more accurate and reliable, as evidenced by the images captured experimentally. For a fibre orientation of 90°, the classic FEM and the FEM-SPH hybrid models exhibit comparable chip formation mechanisms. However, the hybrid model produces slightly better chip morphology. Nevertheless, both models underestimate the thrust and cutting forces. By integrating cohesive elements representing the fibre–matrix interface, the FEM-SPH model provides a better prediction of force calculations and material removal mechanisms. It was also found that the particle size of the SPHs used and the matrix damage variable in the analysis are critical and they can negatively affect the developed force predictions. Overall, this study highlights the promising results of the proposed hybrid model and how it can open up a new direction in simulating machining processes.

## Figures and Tables

**Table 1:** CFRP and epoxy matrix properties [21,31,36,48,49].

Material	Property	Value
Carbon fibre	Elastic constants Longitudinal strength	E_1_ = 294 GPa, E_2_ = E_3_ = 14 GPa GPa, G_23_ = 5.5 GPa*ν*_12_ = *ν*_13_ = 0.2, *ν*_23_ = 0.25G_12_ = G_13_ = 28 GPa X*_t_*= 5.88 GPa, X*_c_*= 3.288 GPa
	Compressive strain failure	0.155
	Compressive yield	594.5 MPa
Epoxy matrix	Elastic constants	E = 2.96 GPa, *ν* = 0.4
Interface	Failure strength fracture Normal strength shear energy	*σ_u_* = 74.7 MPa *τ_max_* = 25 MPa G*^c^*= 0.05 *σ_max_* = 167.5 MPa N/mm^2^

**Table 2:** The thrust force (measured in N/mm) obtained from the FEM-SPH model, FEM, and experimental measurements.

	Trust Force at Samples with Fibre Orientation of 0°	Trust Force at Samples with Fibre Orientation of 90°
Measured experimentally	35.68 N/mm	59.29 N/mm
Calculated using the FEM model	−3.03 N/mm	−4.28 N/mm
Calculated using the hybrid model with D = 0.8	14.2 N/mm	N/A
Calculated using the hybrid model with D = 0.1	16.1 N/mm	24.9 N/mm

## Data Availability

All data presented in this paper that support the findings of this study are included within this paper. Additional data are available from the corresponding author upon reasonable request.
